# Influence Factors on Injury Severity of Traffic Accidents and Differences in Urban Functional Zones: The Empirical Analysis of Beijing

**DOI:** 10.3390/ijerph15122722

**Published:** 2018-12-03

**Authors:** Zhiyuan Sun, Jianyu Wang, Yanyan Chen, Huapu Lu

**Affiliations:** 1 Beijing Key Laboratory of Traffic Engineering, Beijing University of Technology, Beijing 100124, China; sunzhiyuan@bjut.edu.cn (Z.S.); cdyan@bjut.edu.cn (Y.C.); 2 Institute of Transportation Engineering, Tsinghua University, Beijing 100084, China; luhp@tsinghua.edu.cn

**Keywords:** binary logistic regression, classification and regression tree, consistence analysis, injury severity

## Abstract

The objective of this study was to identify influence factors on injury severity of traffic accidents and discuss the differences in urban functional zones in Beijing. A total of 3982 sets of accident data in Beijing were analyzed from the perspective of whole city and different urban functional zones. From the aspects of accident attribute, occurrence time, infrastructure, management status, and environmental condition, the influence factors set of injury severity of traffic accidents in Beijing are set up in this paper, which include 17 influence factors. Based on Pearson’s chi-squared test, factors are preselected. On the basis of binary logistic regression analysis, the impact of the value of influence factors on injury severity of traffic accidents is calibrated. Based on classification and regression tree analysis, the impact of influence factors is analyzed. Through Pearson’s chi-squared test and binary logistic regression analysis, it is found that there are similarities and differences among different urban functional zones. There are two common influence factors, including accident type and cross-section position, and six personalized influence factors, including lighting conditions, visibility, signal control, road physical isolation facility, occurrence period and road type, and the other nine weak influence factors. The results of binary logistic regression analysis and classification and regression tree analysis are basically the same. The factors that should be paid attention to in different urban functional zones and the value of the factors that need special attention are determined by synthesizing two methods.

## 1. Introduction

Road traffic injuries have a huge impact on health security and development. There were 1.25 million road traffic deaths globally in 2013 [1]. In 2015, a total of 187,781 traffic accidents occurred in China, including 58,022 deaths and 199,880 injuries, with a direct economic loss of more than 1 billion RMB [2]. It is very important to study the influencing factors of traffic accidents and eliminate potential accidents.

China has biggest population in the world and has huge area. There are number of variety among different regions. For megacities, there are some differences between different urban functional zones. Generally, the accident cause analysis in the whole city, and the differences among the urban functional zones are often neglected, which can be found by analyzing the statistical data of traffic accidents. Taking Beijing as an example, it can be divided into four parts based on urban master planning. Zone 1 is the capital functional core zone, Zone 2 is the urban functional expansion zone, Zone 3 is the new urban development zone, and Zone 4 is the ecological conservation development zone. According to statistical yearbook of Beijing in 2015, there were 2639 traffic accidents happened, from which 921 were killed and 2619 were injured, there was also recorded a large economic loss, which was approximately 20 million RMB [3]. Differences of traffic accidents in urban functional zones [4] are shown in Table 1. During the analysis of traffic accident causes and factors, we need to pay special attention to the difference between regions based on the overall analysis of the city.

Additionally, considering the factors related to traffic accidents, there are great differences among different urban functional zone. Taking Beijing as the research object, this paper analyses the influence factors on injury severity of traffic accidents, and discusses the difference among the whole city and different urban functional zones.

## 2. Literature Review

### 2.1. Analysis of Traffic Accidents in Beijing

As the capital and one of the largest cites in China, Beijing has a population of 21.7 million people and 5.6 million motor vehicles [3]. Recently, many studies focused on the traffic accidents in Beijing. Yan et al. [5] presented a comprehensive analysis of motor vehicle–bicycle crashes to find the interrelationship of irregular maneuvers, crash patterns, and bicyclist injury severity. Zhao et al. [6] investigated the relative likelihood of pedestrian head injuries based on person, vehicular, and environmental factors. Qiu et al. [7] put forward a novel multi-objective particle swarm optimization-based partial classification method to identify the contributing factors that influence accident severity. Li and Guo [8] developed a sub-distribution hazard regression model for competing risks analysis on traffic accident duration time. Yuan and Chen [9] established a logistic regression model to analyze the significance of main contributing factors of vehicle to vulnerable road user crash. Recently, most of the researchers have studied traffic accidents in Beijing, which are based on influence factors of certain type of accidents.

Most of these studies focus on risk analysis, accident cause mechanism, behavior analysis, etc. Furthermore, these studies are generally small sample studies, since it is difficult to conduct a data survey. Therefore, almost no studies have been conducted on different functional zone. However, one or several types of traffic accidents are difficult to reflect overall characteristics of traffic accidents in Beijing, and the difference among zones is not negligible. In fact, the urban traffic safety activities are based on overall characteristic of urban traffic accidents. This paper is mostly based on the various types of traffic accidents in Beijing, the basis for traffic safety governance activities is put forward for the whole city and different urban functional zones. Traffic safety activities are very important ways to reduce traffic accidents systematically, which include both the planning and design of infrastructure, and the daily traffic management. These contents determine the choice of influence factors of traffic accident.

### 2.2. Influence Factors on Traffic Accidents

Many prior studies have examined the influence factors on traffic accidents. Šliupas [10] discussed the impact of road parameters and surrounding area on traffic accidents. Miškinis and Valuntaite [11] examined the correlation between traffic accidents and driving experience. Kunt et al. [12] considered driver information, vehicle information, weather condition, road surface, etc. Beak et al. [13] dealt the relations between operational method and traffic accidents. Ivan et al. [14] analyzed traffic accidents under low-light conditions. Lu et al. [15] studied the correlation between accident injury severity and potential factors, such as driver factors, environmental factors, vehicle factors, and tunnel factors.

Most of the current studies about the influence factors focus on accident attributes, occurrence time, infrastructure, management status and environmental conditions. However, different influence factors show different impact in different environments. Mathematical models need to be established to calibrate the relationship. Current studies commonly adopt negative binomial regression model [16,17], structural equation model [18], linear and multiple regressions model [10], random effects model [19], hypothesis testing model [13], multiple logistic regression [20], ordered logit model [15], etc.

In previous publications, the traffic safety depends on the integrated and complex relationship between various components [21]. For example:

A. human factor: the psychology of the vehicle’s driver, pedestrian, etc.;

B. traffic flow: the traffic, the vehicle, signal control mode, etc.;

C. road infrastructure: road type, road line style, central isolation facility, etc.;

D. environmental condition: road safety attribute, lighting condition, etc.

In the study of influence factors on injury severity of traffic accidents in Beijing, a set of influence factors is established from multiple aspects. Further screening of influence factors and careful analysis of core factors are carried out.

## 3. Data

Based on the statistical data of traffic accidents in Beijing, a set of influence factors on injury severity of traffic accidents in Beijing is set up. *Y* indicates injury severity of traffic accident. *X_i_* indicates the independent variable that has a significant impact on injury severity of traffic accidents. The set of influence factors includes five aspects:

*A.* Accident attribute, including accident type *X*_1_;

*B.* Time of occurrence, including day of the week *X*_2_ and time interval *X*_3_;

*C.* Infrastructure, including cross-section position *X*_4_, central isolation facility *X*_5_, physical isolation facility *X*_6_, pavement condition *X*_7_, pavement structure *X*_8_, intersections type *X*_9_, road line style *X*_10_, and road type *X*_11_;

*D.* Management status, including road safety attribute *X*_12_ and signal control mode *X*_13_; and

*E.* Environment condition, including weather *X*_14_, visibility *X*_15_, lighting condition *X*_16_, and road surface condition *X*_17_.

Based on the investigation of injuries in Beijing from 2014 to 2015, 3982 data points are selected. These data have excluded abnormal data, such as imperfect records and obvious error. The definitions and descriptive statistics of *Y* and *X*_i_ are shown in Table 2.

From the perspective of accident attribute, there are statistically analysis of the traffic accidents in whole city and different functional zones of Beijing. It is easy to find that there are certain differences among different urban functional zones. Differences of traffic accident type in urban functional zone are shown in Table 3. Differences of other traffic characteristics in urban functional zone [4] are shown in Table 4.

## 4. Methods

With 3982 sets of accident data in Beijing, considering the perspective of whole city and urban functional zones, the factors that really affect injury severity of traffic accidents can be screened from a series of candidate influence factors. First, based on Pearson’s chi-squared test, the correlation between severity and influencing factors and the influence factors are selected to reduce the difficulty of the latter analysis. Secondly, based on binary logistic regression analysis, the influence of various factors is studied. Finally, based on classification and regression tree analysis, the influence degree of various factors is studied.

### 4.1. Pearson’s Chi-Squared Test

The relationship between *Y* and *X_i_* (I = 1, 2, …, 17) is studied to realize preliminary screening of influence factors. Collating data through the contingency table, where columns indicate *X_i_*, and rows indicate *Y*. If *X_i_* has *h* levels, and *Y* has *l* levels, then the table is called *h* × *l* contingency table. It is considered that the chi-square statistic is of significance at the 0.05 level.

### 4.2. Binary Logistic Regression Analysis

The influence factors preliminarily screened by Pearson’s chi-squared test should be further selected, and the effects of the selected factors on injury severity of traffic accidents should be determined. This analysis process can be realized by binary logistic regression analysis (BLR).

The probability of the occurrence of traffic accident severity is:

(1) $$P(Y|X_{1},X_{2},\cdots ,X_{17})=\frac{1}{1+e^{\left(\alpha +\sum_{i=1}^{17}\beta_{i}X_{i}\right)}}$$

where *α* is constant, and *β_i_* is the parameter of the independent variable.

Further transformed into logarithmic form:

(2) $$\ln\frac{P}{1+P}=\alpha +\sum_{i=1}^{17}\beta_{i}X_{i}$$

All the factors that affect the severity of accidents should be screened, in order to find out the factors that have a significant impact on the severity of traffic accidents. A significance level of 0.05 of screening rule is suggested.

### 4.3. Classification and Regression Tree Analysis

Classification and regression tree (CART) is a learning method for conditional probability distribution of output random variables under given input conditions. CART dichotomizes each characteristic. After the selection of the best binary characteristics and the best binary eigenvalue and dichotomy, the binary tree is generated, and CART algorithm is implemented by pruning. Classification CART tree selects Gini coefficient criterion for feature selection.

In a classification problem, supposing there a *K* classes, the probability of the sample point belonging to class *k* is *p_k_*, then the Gini index of the probability distribution is defined as:

(3) $$Gini\left(p\right)=\sum_{k=1}^{K}p_{k}\left(1-p_{k}\right)=\left(p_{1}+p_{2}+\cdots +p_{k}\right)-\sum_{k=1}^{K}p_{k}^{2}=1-\sum_{k=1}^{K}p_{k}^{2}$$

Set *C_k_* as the subset of class *k* of sample *D*, then the Gini index is:

(4) $$Gini\left(D\right)=1-\sum_{k=1}^{K}{\left(\frac{\left|C_{k}\right|}{\left|D\right|}\right)}^{2}$$

Supposing the condition *A* divides the sample *D* into two data subsets *D*_1_ and *D*_2_, then the Gini index of the sample *D* under condition *A* is:

(5) $$Gini\left(D,A\right)=\frac{\left|D_{1}\right|}{D}Gini\left(D_{1}\right)+\frac{\left|D_{2}\right|}{D}Gini\left(D_{2}\right)$$

The Gini index also indicates the uncertainty of samples. The importance of the most influential factors is converted to 100%, and that of other factors is converted to percentage in turn. It is considered that the importance of standardization is meaningful at 20% levels.

## 5. Findings

### 5.1. Pearson’s Chi-Squared Test

Based on Pearson’s chi-squared test, the correlation between severity and influence factors are studied in whole city and different urban functional zones. The test value of the chi-square statistics is shown in Table 5.

#### 5.1.1. Whole City

Accident type, time interval, cross-section position, physical isolation facility, pavement condition, intersections type, road line style, road type, signal control mode, visibility, and lighting conditions are 11 factors that are closely connected with severity. In the latter analysis, these 11 factors should be considered.

#### 5.1.2. Zone 1–4

For Zone 1, accident type, cross-section position, pavement condition, and road type are four factors that are closely connected with severity.

For Zone 2, accident type, time interval, cross-section position, pavement structure, intersections type, road safety attribute, weather, and visibility are eight factors that are closely connected with severity.

For Zone 3, accident type, time interval, central isolation facility, physical isolation facility, road line style, road type, signal control mode, visibility, and lighting conditions are nine factors that are closely connected with severity.

For Zone 4, accident type, time interval, cross-section position, road type, and lighting condition are five factors that are closely connected with severity.

**Figure 1:**
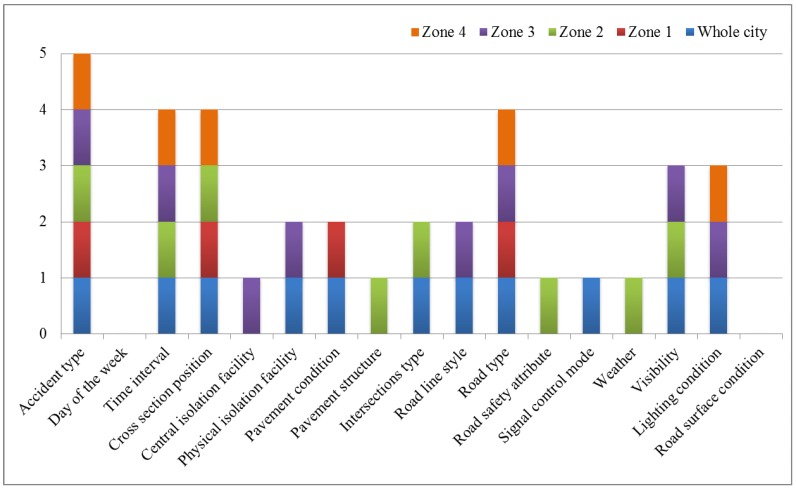
The statistical results of Pearson’s chi-squared test.

As shown in Figure 1, the following results can be found: accident type, time interval, cross-section position, and road type appeared no less than four times; central isolation facility, physical isolation facility, pavement condition, pavement structure, intersections type, road line style, road safety attribute, signal control mode, weather, visibility, and lighting conditions appeared, but less than four times; day of the week and road surface condition did not appear.

### 5.2. Binary Logistic Regression Analysis

Based on BLR, the influence factors on injury severity of traffic accidents are studied in whole city and different urban functional zones. The results of BLR are shown in Table 6.

#### 5.2.1. Whole City

The probability of death accident of accident type 1, 2, 3, and 4 are, separately, 1.598 times, 4.422 times, 3.353 times, and 2.401 times that of accident type 5. The top two probabilities of death accident are accident types 2 and 3.

The probability of death accident of cross-section position 1, 2, 3, 4, 5, and 6 are, separately, 0.794 times, 0.794 times, 0.794 times, 2.105 times, 0.614 times, and 2.858 times that of cross-section position 7. The top two probabilities of death accident are cross-section positions 6 and 4.

The probability of death accident of physical isolation facility 1, 2, and 3 are, separately, 1.059 times, 0.846 times, and 1.334 times that of physical isolation facility 4. The top two probabilities of death accident are physical isolation facilities 3 and 1.

The probability of death accident of road type 1, 2, 3, 4, and 5 are, separately, 2.231 times, 1.091 times, 0.994 times 1.170 times, and 1.502 times that of road type 6. The top two probabilities of death accident are road types 1 and 5.

The probability of death accident of signal control mode 1 and 2 are, separately, 0.785 times and 0.785 times that of signal control mode 3. The top two probabilities of death accident are signal control modes 2 and 3.

The probability of death accident of visibility 1, 2, and 3 are, separately, 0.590 times, 1.028 times and 1.273 times that of visibility 4. The top two probabilities of death accident are visibilities 3 and 2.

The probability of death accident of lighting conditions 1, 2, 3, and 4 are, separately, 1.020 times, 0.914 times, 2.162 times, and 2.044 times that of lighting condition 5. The top two probabilities of death accident are lighting conditions 3 and 4.

#### 5.2.2. Zone 1–4

Factors affecting the probability of death accident differ from zone 1 to zone 4. The top probability of death accident relating to each factor can was shown in Table 4 and the analysis process is the same to the whole city in order to find the most influencing factors.

**Figure 2:**
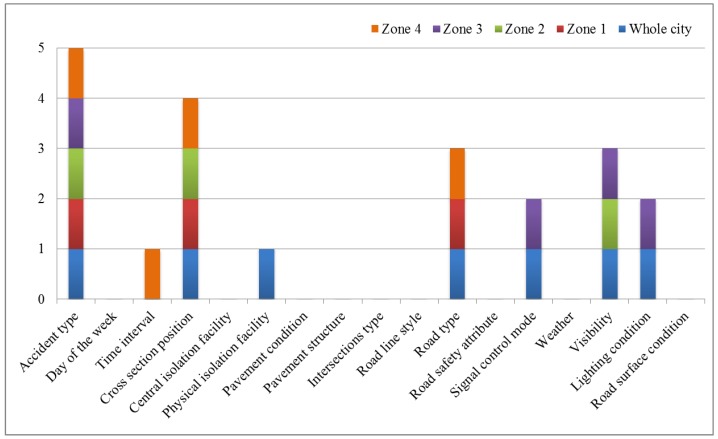
The statistical results of BLR.

By BLR, the influencing factors are further screened out. As shown in Figure 2, the following results can be found: accident type and cross-section position appeared no less than four times; lighting condition, visibility, signal control mode, physical isolation facility, central isolation facility, time interval, and road type appeared, but less than four times; day of the week, pavement condition, pavement structure, intersections type, road line style, road safety attribute, weather, road surface condition did not appear.

Compared with the results of Pearson’s chi-squared test, there are some differences. The number of factors that appear no less than four times decreased by 2; the number of the factors that appeared but less than four times decreased by 5; and the factors that do not appear increased by 7.

### 5.3. Classification and Regression Tree Analysis

Based on CART, the influence degree of various factors on injury severity of traffic accidents is studied in whole city and different urban functional zones. CART considers that the importance of standardization is meaningful at the 20% level. The results of CART are shown in Table 7.

#### 5.3.1. Whole City

According to the magnitude of accident severity, the influence factors are sorted in turn: lighting condition, accident type, road type, visibility, signal control mode, time interval, physical isolation facility, cross-section position, pavement condition, road line style, and intersections type. For the top five, the importance of standardization is more than 20%.

#### 5.3.2. Zone 1

According to the magnitude of accident severity, the influence factors are sorted in turn: accident type, cross-section position, road type, and pavement condition. The importance of standardization of all factors is more than 20%.

#### 5.3.3. Zone 2

According to the magnitude of accident severity, the influence factors are sorted in turn: accident type, cross-section position, visibility, weather, time interval, and pavement structure. For the top five, the importance of standardization is more than 20%.

#### 5.3.4. Zone 3

According to the magnitude of accident severity, the influence factors are sorted in turn: lighting condition, road type, visibility, accident type, physical isolation facility, signal control mode, central isolation facility, time interval, and road line style. For the top seven, the importance of standardization is more than 20%.

#### 5.3.5. Zone 4

According to the magnitude of accident severity, the influence factors are sorted in turn: accident type, time interval, cross-section position, road type, and lighting condition. The importance of standardization of all factors is more than 20%.

## 6. Discussion

### 6.1. Consistence Analysis of BLR and CART

BLR and CART analyze the characteristics of influence factors from different angles. It is necessary to discuss the consistency of the two methods. As shown in Table 8, the conclusions of the two methods are basically the same.

### 6.2. Comparative Analysis of Influencing Factors

**Figure 3:**
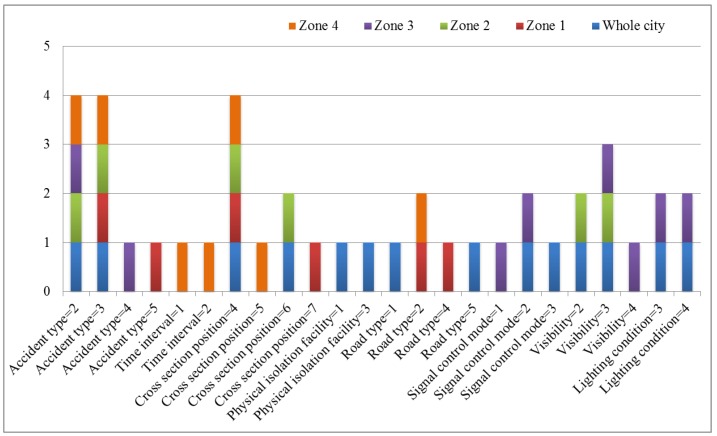
Comparative analysis of influencing factors.

Based on the results of BLR, the influence factors that appear more no less than four times are defined as common influence factors, those that appear but less than 4 times as personalized influence factors, and those that don’t appear as weak influence factors. Further analysis of the difference among whole city and different function zones is shown in Figure 3.

#### 6.2.1. Accident Type

As shown in the previous study by Al-Ghamdi [22], accident type is a common influence factor, and needs attention when the value is 2 or 3. In the whole city, zone 2 and zone 4 should pay attention to accident types 2 and 3. In zone 3, accident type 4 should be focused besides accident type 2. In zone 1, accident type 5 should be focused besides accident type 3.

#### 6.2.2. Time Interval

Time interval is a personalized influence factor. Only in zone 4, time intervals 1 and 2 should be considered. Similar results were found by Kim [23] that morning rush hour (between 06:00 and 09:59 a.m.) made an increase of fatal probability.

#### 6.2.3. Cross-section Position

Cross-section position is a common influence factor, the value 4 needs more attention. In the whole city and zone 2, cross-section positions 4 and 6 need focus, in zone 1 cross-section positions 4 and 7, while in zone 4 cross-section positions 4 and 5. Several previous studies [24,25,26] indicated a similar result that cycling on sidewalk was more dangerous than on the road.

#### 6.2.4. Physical Isolation Facility

Physical isolation facility is a personalized influence factor. Only in whole city physical isolation facility 1 and 3 should be paid attention to. Osman et al. [27] also showed similar result that lack of access-control increased the possibility of serious injury.

#### 6.2.5. Road Type

Road type is a personalized influence factor, with great variety. In the whole city road types 1 and 5 should be considered, in zone 1 road types 2 and 4, while in zone 4 only road type 2. Previous study [27] also showed that urban principal arterial could contribute to injury severity.

#### 6.2.6. Signal Control Mode

Signal control mode is a personalized influence factor. In the whole city signal control modes 2 and 3 need more attention, and in zone 3 signal control modes 1 and 2, which was similar to the result reported by Osman et al. [27] that signalized control made for a lower likelihood of serious injury in comparison with non-signalized control.

#### 6.2.7. Visibility

Visibility is a personalized influence factor. In whole city and zone 2 visibility 2 and 3 need to be paid attention to, while in zone 3 visibility 3 and 4. Klop and Khattak’s [28] study showed that fog increased injury severity partially because the inclement weather reduced visibility.

#### 6.2.8. Lighting Condition

Lighting condition is a personalized influence factor. The distribution of index is more concentrated. In whole city and zone 3, lighting conditions 3 and 4 need more attention. A similar result was found by Ivan et al. [14] that low lighting conditions significantly influenced accident occurrence. 

### 6.3. Comparative Analysis of Urban Functional Zone

Integrating the conclusion of BLR and CART, the features of different urban functional zone are explored.

#### 6.3.1. Whole City

The factors that should be focused on in whole city include: lighting condition, accident type, road type, visibility and signal control mode.

In particular, attention should be paid to the following situation: the value of lighting condition is 3 or 4; the value of accident type is 2 or 3; the value of road type is 1 or 5; the value of visibility is 3 or 2; the value of signal control mode is 2 or 3.

#### 6.3.2. Zone 1

The factors that should be focused on in zone 1 include: accident type, cross-section position, road type, and pavement condition.

In particular, attention should be paid to the following situation: the value of accident type is 3 or 5; the value of cross-section position is 7 or 4; the value of road type is 4 or 2.

Zone 1 is the center of city, with the main characteristics of high population density and high branch road density. The value of accident type, cross-section position and road type are connected with pedestrians and motor vehicles. 

#### 6.3.3. Zone 2

The factors that should be focused on in zone 2 include: accident type, cross-section position, visibility, weather, and time interval.

In particular, attention should be paid to the following situation: the value of accident type is 2 or 3; the value of cross-section position is 6 or 4; the value of road type is 3 or 2.

Zone 2 surrounds the city center, with the main characteristic that arterial roads of expressway have a relatively high density. The value of accident type, cross-section position, and road type are connected with vehicle traffic. 

#### 6.3.4. Zone 3

The factors that should be focused on in zone 3 include: lighting condition, road type, visibility, accident type, physical isolation facility, signal control mode, and central isolation facility.

In particular, attention should be paid to the following situation: the value of lighting condition is 3 or 4; the value of visibility is 3 or 4; the value of accident type is 2 or 4; the value of signal control mode is 2 or 1.

Zone 3 has a certain distance from the city center, with the main characteristic that high-grade roads have a relatively high density. Therefore, it is different from zone 1 and zone 2 that the value of some traffic facilities and environmental indicators, such as physical isolation facility, lighting conditions, accident type, signal control mode, and visibility are connected with high-grade roads.

#### 6.3.5. Zone 4

The factors that should be focused on in zone 4 include: accident type, time interval, cross-section position, road type, and lightning condition.

In particular, attention should be paid to the following situation: the value of the accident type is 3 or 2; the value of the time interval is 1 or 2; the value of the cross-section position is 5 or 4; the value of the road type is 2.

Zone 4 is the suburban area of the city, with the main characteristics of low population density and nighttime vehicle crossing. Therefore, the value of accident type, road type, time interval, and cross-section position are connected with low population density and nighttime vehicle crossing. For example, the time interval indicates nighttime.

## 7. Conclusions

Taking Beijing as an example, 3982 sets of accident data were analyzed from the perspective of whole city and different urban functional zones. The influence factors set of injury severity of traffic accidents were set up from the aspects of accident attribute, occurrence time, infrastructure, management status, and environmental condition. These factors are preselected based on Pearson’s chi-squared test. The impact of the value of these influence factors on injury severity is calibrated based on binary logistic regression analysis. Additionally, the impact of influence factors is analyzed based on classification and regression tree analysis.

It is found that there are similarities and differences among different urban functional zones. There are two common influence factors, including accident type and cross-section position, and six personalized influence factors, including lighting conditions, visibility, signal control, road physical isolation facility, occurrence period, and road type, and nine other weak influence factors. The results of binary logistic regression analysis and classification and regression tree analysis are basically the same. The factors that should be paid attention to in different urban functional zones and the value of the factors that need special attention are determined by synthesizing two methods. It can be concluded that the difference of influence factors on injury severity in different zones is connected with different zones’ attribute.

## Figures and Tables

**Table 1:** Differences of traffic accident in urban functional zone

Zone	Traffic Accidents (unit)	Death Toll (unit)	Economic Loss (1000 yuan)
Zone 1: Dongcheng District Xicheng District	146	25	80.8
Zone 2: Chaoyang District Fengtai District Shijingshan District Haidian District	857	296	644.0
Zone 3: Fangshan District Tongzhou District Shunyi Distric Changping District Daxing Distric	1318	457	991.0
Zone 4: Huairou District Pinggu District Miyun District Yanqing District Mentougou District Yizhuang District	294	137	339.5

**Table 2:** Definitions and descriptive statistics.

Variables	Definition	Mean Value	Standard Deviation
Severity	1 = Death accident; 2 = Injury without death	1.58	0.49
Accident attribute			
Accident type	1 = 2 vehicles and above, without pedestrians or motor vehicles; 2 = 1 vehicle, without pedestrians or motor vehicles; 3 = 2 vehicles and above, with pedestrians or motor vehicles; 4 = 1 vehicle, with pedestrians or motor vehicles; 5 = Only pedestrians or motor vehicles	2.79	1.45
Time of occurrence			
Day of the week	1 = Monday; 2 = Tuesday; 3 = Wednesday; 4 = Thursday; 5 = Friday; 6 = Saturday; 7 = Sunday	4.04	1.96
Time interval	1 = 0:00–6:00; 2 = 6:00–12:00; 3 = 12:00–18:00; 4 = 18:00–24:00	2.75	1.05
Infrastructure			
Cross-section position	1 = Motorized Lane; 2 = Non-motorized Lane; 3 = Mixed Lane; 4 = Sidewalk; 5 = Pedestrian crossing; 6 = Emergency parking area; 7 = Others	1.88	1.67
Central isolation facility	1 = Green area; 2 = Concrete retaining; 3 = Isolated piers (columns); 4 = Others	3.13	1.18
Physical isolation facility	1 = No isolation; 2 = Central isolation; 3 = Isolation between motor and non-motor vehicle; 4 = Center of isolation and isolation between motor and non-motor vehicle	1.71	0.73
Pavement condition	1 = Good condition; 2 = Under construction; 3 = Concave-convex; 4 = Collapse; 5 = Barricade	1.03	0.26
Pavement structure	1 = Bitumen; 2 = Cement; 3 = Sand or stone; 4 = Soil road; 5 = Others	1.03	0.24
Intersections type	1 = Intersection; 2 = General section; 3 = Others	1.73	0.51
Road line style	1 = Straight; 2 = Curve	1.04	0.20
Road type	1 = Highway; 2 = Urban Expressway; 3 = Urban trunk road; 4 = Other urban roads; 5 = High grade road; 6 = Others	3.87	1.28
Management status			
Road safety attribute	1 = Normal road; 2 = Section with lurking peril managed; 3 = Section with lurking peril being managed; 4 = Section with lurking peril but not managed; 5 = Others	2.23	1.77
Signal control mode	1 = No signal; 2 = Other security facilities; 3 = Signal	1.83	0.68
Environment condition			
Weather	1 = Sunny; 2 = Cloudy; 3 = Rainy; 4 = Snowy; 5 = Foggy; 6 = Windy; 7 = Dust; 8 = Hailstones; 9 = Others	1.22	0.64
Visibility	1 = Under 50m; 2 = 50–100 m; 3 = 100–200 m; 4 = More than 200m	3.03	1.03
Lighting condition	1 = Daytime; 2 = Night with street lamp lighting; 3 = Night without street lamp lighting; 4 = Dawn; 5 = Dust	1.72	0.91
Road surface condition	1 = Dry; 2 = Damp; 3 = Ponding; 4 = Overflowing; 5 = Ice and snow; 6 = Others	1.15	0.73

**Table 3:** Differences of traffic accident type in urban functional zone.

Zone	Severity		Accident Type				
	*Y* = 1	*Y* = 2	*X*_1_ = 1	*X*_1_ = 2	*X*_1_ = 3	*X*_1_ = 4	*X*_1_ = 5
Whole city	42.4%	57.6%	34.9%	9.0%	3.1%	48.4%	4.5%
Zone 1	28.1%	71.9%	23.1%	5.8%	2.1%	60.3%	8.7%
Zone 2	39.5%	60.5%	33.1%	8.1%	3.9%	50.5%	4.5%
Zone 3	43.9%	56.1%	36.4%	9.2%	3.1%	46.5%	4.9%
Zone 4	49.3%	50.7%	38.6%	11.3%	2.2%	45.5%	2.4%

**Table 4:** Differences of other traffic characteristics in urban functional zone.

Zone	Permanent Resident Population (Ten Thousand)	Permanent Resident Population per Square Kilometer	Car Ownership	Car Ownership per 1000 People
Whole city	2170.5	1323	5,349,989	246.5
Zone 1	220.3	23,845	933,336	423.7
Zone 2	1062.5	8327	2,599,993	244.7
Zone 3	696.9	1107	1,417,004	203.3
Zone 4	190.8	218	399,656	209.4

**Table 5:** Pearson’s chi-squared test.

Variables	Whole City	Zone 1	Zone 2	Zone 3	Zone 4
Accident attribute					
Accident type	0.000	0.000	0.000	0.000	0.000
Time of occurrence					
Day of the week	-	-	-	-	-
Time interval	0.000	-	0.037	0.003	0.019
Infrastructure					
Cross-section position	0.000	0.007	0.000	-	0.025
Central isolation facility	-	-	-	0.000	-
Physical isolation facility	0.030	-	-	0.006	-
Pavement condition	0.012	0.028	-	-	-
Pavement structure	-	-	0.031	-	-
Intersections type	0.012	-	0.031	-	-
Road line style	0.021	-	-	0.006	-
Road type	0.000	0.009	-	0.000	0.003
Management status					
Road safety attribute	-	-	0.037	-	-
Signal control mode	0.017	-	-	0.000	-
Environment condition					
Weather	-	-	0.013	-	-
Visibility	0.000	-	0.008	0.000	-
Lighting condition	0.000	-	-	0.000	0.014
Road surface condition	-	-	-	-	-

“-” indicates that the chi-square statistics are meaningless.

**Table 6:** Binary logistic regression analysis.

Variables	Whole City		Zone 1		Zone 2		Zone 3		Zone 4	
	Sig.	Exp(B)	Sig.	Exp(B)	Sig.	Exp(B)	Sig.	Exp(B)	Sig.	Exp(B)
Accident attribute										
Accident type	0.000		0.002		0.000		0.000		0.000	
1		1.598		0.072		0.696		5.401		1.056
2		4.422		0.337		1.927		13.304		4.391
3		3.353		5.134		1.718		6.567		5.523
4		2.401		0.424		1.272		6.665		1.781
5		#		#		#		#		#
Time of occurrence										
Time interval	-		-		-		-		0.050	
1										2.586
2										1.223
3										1.112
4										#
Infrastructure										
Cross-section position	0.001		0.031		0.001		-		0.048	
1		0.794		0.468		0.722				0.470
2		0.584		0.140		0.380				0.332
3		0.723		0.096		0.514				0.768
4		2.105		0.738		1.195				1.239
5		0.614		0.414		0.325				2.171
6		2.858		/		2.884				/
7		#		#		#				#
Central isolation facility	-		-		-		0.000		-	
1								1.177		
2								2.261		
3								2.355		
4								#		
Physical isolation facility	0.007		-		-		0.000		-	
1		1.059						0.877		
2		0.846						0.455		
3		1.334						1.349		
4		#						#		
Road type	0.000		0.024		-		0.001		0.004	
1		2.231		/				2.326		4.375
2		1.091		0.544				1.291		6.900E8
3		0.994		0.298				1.836		0.494
4		1.170		#				1.738		0.798
5		1.502		/				1.859		1.015
6		#		/				#		#
Management status										
Signal control mode	0.001		-		-		0.004		-	
1		0.785						1.003		
2		1.060						1.428		
3		#						#		
Environment condition										
Visibility	0.000		-		0.004		0.000		-	
1		0.590			0.143	0.662		0.489		
2		1.028			0.074	1.326		0.953		
3		1.273			0.004	1.564		1.336		
4		#				#		#		
Lighting condition	0.000		-		-		0.000		-	
1		1.020						1.227		
2		0.914						1.108		
3		2.162						3.153		
4		2.044						2.390		
5		#						#		

“-” indicates that the significance test is more than 0.05, and it is meaningless. “/” indicates that there are no data. “#” indicates the reference value of Exp (B).

**Table 7:** Classification and regression tree analysis.

Variables	Whole City	Zone 1	Zone 2	Zone 3	Zone 4
Accident attribute					
Accident type	80.8%	100.0%	100%	55.4%	100.0%
Time of occurrence					
Day of the week	-	-	-	-	-
Time interval	*	-	37.5%	*	62.9%
Infrastructure					
Cross-section position	*	43.1%	68.3%	-	58.2%
Central isolation facility	-	-	-	38.6%	-
Physical isolation facility	*	-	-	47.2%	-
Pavement condition	*	31.1%	-	-	-
Pavement structure	-	-	*	-	-
Intersections type	*	-	*	-	-
Road line style	*	-	-	*	-
Road type	41.8%	40.3%	-	84.7%	53.4%
Management status					
Road safety attribute	-	-	*	-	-
Signal control mode	25.2%	-	-	44.0%	-
Environment condition					
Weather	-	-	42.0%	-	-
Visibility	28.8%	-	55.3%	62.9%	-
Lighting condition	100.0%	-	-	100.0%	36.5%
Road surface condition	-	-	-	-	-

“-” indicates that the chi-square statistics are meaningless, and classification and regression tree analysis is not carried out. “*” indicates that the value is below 20%.

**Table 8:** Consistence analysis of BLR and CART.

Zone	BLR	CART
Whole city	Accident type, cross-section position, physical isolation facility, road type, signal control mode, visibility, lighting condition	Lighting condition, accident type, road type, visibility, signal control mode
Zone 1	accident type, cross-section position, road type	Accident type, cross-section position, road type, pavement condition
Zone 2	Accident type, cross-section position, visibility	Accident type, cross-section position, visibility, weather, time interval
Zone 3	Accident type, signal control mode, visibility, lighting condition	lighting condition, road type, visibility, Accident type, physical isolation facility, signal control mode, central isolation facility
Zone 4	Accident type, time interval, cross-section position, road type	Accident type, time interval, cross-section position, road type, lighting condition
